# CD73 Rather Than CD39 Is Mainly Involved in Controlling Purinergic Signaling in Calcified Aortic Valve Disease

**DOI:** 10.3389/fgene.2019.00604

**Published:** 2019-07-25

**Authors:** Igor Kudryavtsev, Maria Serebriakova, Ekaterina Zhiduleva, Patimat Murtazalieva, Vladislav Titov, Anna Malashicheva, Anastasya Shishkova, Daria Semenova, Olga Irtyuga, Dmitry Isakov, Lubov Mitrofanova, Olga Moiseeva, Alexey Golovkin

**Affiliations:** ^1^Institution of Experimental Medicine, St. Petersburg, Russia; ^2^Far Eastern Federal University, Vladivostok, Russia; ^3^Almazov National Medical Research Centre, St. Petersburg, Russia; ^4^Pavlov First Saint Petersburg State Medical University, St. Petersburg, Russia

**Keywords:** calcified aortic stenosis, T-cells (or lymphocytes), purinergic signaling, calcification, CD39 and CD73 expression

## Abstract

The study aimed to compare composition of peripheral blood T-cell subsets and assess their surface expression of CD39 and CD73 ectonucleotidases in patients with severe and moderate aortic stenosis (AS) as well as to evaluate involvement of T-cell-mediated immune processes in valve calcification. The study was performed with 38 patients suffering from severe calcified aortic stenosis (SAS), 33 patients with MAS, and 30 apparently healthy volunteers (HVs). The relative distribution and percentage of T-cell subsets expressing CD39 and CD73 were evaluated by flow cytometry. T helper (Th) and cytotoxic T-cell subsets (Tcyt) were identified by using CD3, CD4, and CD8 antibodies. Regulatory T cells (Tregs) were characterized by the expression of CD3, CD4, and high IL-2R alpha chain (CD25high) levels. CD45R0 and CD62L were used to assess differentiation stage of Th, Tcyt, and Treg subsets. It was found that MAS and SAS patients differed in terms of relative distribution of Tcyt and absolute number of Treg. Moreover, the absolute number of Tcyt and terminally differentiated CD45RA-positive effector T-cells (TEMRA) subset was significantly higher in SAS vs. MAS patients and HVs. However, the absolute and relative number of naïve Th and the absolute number of Treg were significantly higher in MAS vs. SAS patients; the relative number of naïve Tregs was significantly (*p* < 0.01) decreased in SAS patients. It was shown that CD73 expression was significantly higher in SAS vs. MAS patients noted in all EM, CM, TEMRA, and naïve Th cell subsets. However, only the latter were significantly increased (*p* = 0.003) in patients compared with HVs. SAS vs. MAS patients were noted to have significantly higher percentage of CD73+ EM Tcyt (*p* = 0.006) and CD73+ CM Tcyt (*p* = 0.002). The expression of CD73 in patients significantly differed in all three Treg populations such as EM (*p* = 0.049), CM (*p* = 0.044), and naïve (*p* < 0.001). No significant differences in CD39 expression level was found in MAS and SAS patients compared with the HV group. Overall, the data obtained demonstrated that purinergic signaling was involved in the pathogenesis of aortic stenosis and calcification potentially acting *via* various cell types, wherein among enzymes, degrading extracellular ATP CD73 rather than CD39 played a prominent role.

## Introduction

A significant number of studies have been recently published evidencing about an important role played by the receptors for extracellular ATP and its metabolites as well as enzymes involved in regulating their metabolism in vascular and heart valve calcification (Fish et al., 2013; Towler, 2017; Dou et al., 2018; Golovkin et al., 2018; Kaniewska-Bednarczuk et al., 2018). Previously, it was shown that various receptor isotypes specific to extracellular adenosine (P1 family) and ATP (Р2Х and Р2Y families) were found on smooth muscle cells, the major cell type involved in formation of calcium depositions in the vascular wall (Fish et al., 2013). Nucleotides, in turn, present in extracellular space able to activate P1 and P2 purinergic receptors, are metabolized by a several enzymes acting on ATP and its derivatives. In particular, CD39 (E-NTPDase1—ectonucleoside triphosphate diphosphohydrolase 1) cleaves ATP to two AMPs and two phosphate molecules; the latter is now considered as one of the major cues promoting tissue hydroxyapatite deposition (Fish et al., 2013). Another phosphate molecule is generated by CD73 (Ecto5′NTase—ecto-5′-nucleotidase) converting AMP to adenosine (Fish et al., 2013). Inorganic pyrophosphate (PPi) formed during AMP synthesis mediated by ectonucleotide pyrophosphatase/phosphodiesterase (ENPP) represents a potential inhibitor of hydroxyapatite deposition and subsequent tissue calcification (Fish et al., 2013).

In connection with this, investigating the expression of molecules involved in ATP metabolism during vascular and heart valve calcification potentially underlying its pathogenesis is of high priority.

Furthermore, morphology and biochemical examination of the pig aortic valves demonstrated that CD39 and CD73 were upregulated in both endothelial and interstitial cells (Kaniewska et al., 2014). Culturing endothelial and interstitial cell lines added with nucleotides revealed that CD73 product formation was accelerated in interstitial cells compared with endothelial cells, whereas CD39 activity showed an opposite pattern (Kaniewska et al., 2014).

In addition, CD39 and CD73 are widely expressed by immune cells (Bono et al., 2015; Borg et al., 2017; Zhao et al., 2017a). Purinergic regulation plays a crucial role in T-cell functioning. For instance, ATP is involved in controlling regulatory T-cell differentiation and functional activity. Moreover, low ATP level activates whereas its high amount and prolonged P2X7 stimulation trigger pore formation and T-cell apoptosis (Trabanelli et al., 2012). In turn, adenosine exhibits opposite effects on T cells by suppressing functional activity of some T-cell subsets primarily targeting Th17 cells (Faas et al., 2017) but activating regulatory T cells. Moreover, down-modulated IL-2 production is solely mediated by adenosine resulting in suppressed proliferation and differentiation of naïve T cells towards Th1 and Th2 cells in lymphoid tissues (Csóka et al., 2008). Apart from that, adenosine may block TCR signaling after elevating cAMP level *via* A2A receptor stimulation (Stagg and Smyth, 2010). Meanwhile, A2A receptor has been identified as the major anti-inflammatory adenosine receptor associated with T cells (Ohta and Sitkovsky, 2001).

Thus, a role played solely by nucleotides as well as purine receptors found in connective tissue cells and lymphocytes egressing from the circulation seems to be important in the pathogenesis of great vessel and heart valve calcification. Moreover, detection of activated T cells in peripheral blood correlating with the level of aortic valve calcification additionally pointed at the close relationship between circulating immune cells and processes occurring in an inflamed aortic tissue (Winchester et al., 2011). In addition, a number of highly differentiated CD3+CD8+ T cells was increased in these patients, whereas a high number of mature CD28-negative cytotoxic T cells was found microscopically in foci of calcification (Winchester et al., 2011).

Thus, the current study was aimed to compare composition of peripheral blood T-cell subsets and assess their surface expression of CD39 and CD73 ectonucleotidases in patients with severe and moderate aortic stenosis (AS) as well as to evaluate involvement of T-cell-mediated immune processes in valve calcification.

## Materials and Methods

The current study was performed with 38 patients suffering from severe calcified aortic stenosis (SAS) undergoing surgical heart valve replacement and 33 patients with moderate AS (MAS) undergoing nonsurgical treatment. Surgical and nonsurgical treatment and follow-up were performed at the Almazov National Medical Research Centre.

All patients underwent comprehensive two-dimensional and Doppler transthoracic echocardiography by using Vivid 7.0 system (GE, USA), according to the current ECHO guidelines. The criteria for severity of aortic valve stenosis included aortic valve area (AVA, cm^2^) calculated by using continuity equation; AVA indexed for body surface area (AVA/BSA, cm^2^/m^2^); and mean transvalvular pressure gradient and peak aortic jet velocity (Vmax).

A multislice spiral computed tomography with Agatston calcium scoring was performed to assess calcium deposits in the heart valves of MAS patients (Falk et al., 2017), reaching 993 (456; 1,968) and 886 (492; 1,229) in males and females, respectively.

Clinical characteristics of patients are presented in **Table 1**.

**Table 1:** Clinical and hematological characteristics of subjects in various groups.

	Severe stenosis (*n* = 38)	Moderate stenosis (*n* = 33)	Healthy volunteers (*n* = 30)
Age, years	62.5 (57; 66)^	63 (56; 67)^	53 (52; 58)
Male/female ratio	23/15	17/16	18/12
BMI (body mass index), kg/m^2^	27.9 (25.3; 33.1)	28.1 (15.7; 31.9)	
Arterial hypertension, %	30 (78.9%)	29 (87.9%)	
Diabetes mellitus, %	16 (42.1%)	6 (18.2%)	
Ischemic heart disease	16 (42.1%)	7 (21.2%)	
Mean aortic valve gradient, mm Hg	48.3 (44.0; 54.9)*	20.0 (14.0; 27.0)*	
Vmax, m/s	4.56 (4.30; 5.03)*	2.90 (2.47; 3.30)*	
AVA, cm^2^	0.78 (0.70; 0.80)*	1.40 (1.00; 1.70)*	
Left ventricle mass index, g/m^2^	179 (145; 274)*	128 (106; 158)*	
Ejection fraction, %	63 (46; 67)	65 (58; 67)	
C-reactive protein, mg/L	3.49 (1.15; 6.69)	1.90 (1.38; 3.44)	
Cholesterol, mmol/L	4.63 (4.21; 6.21)	5.24 (4.49; 5.79)	
WBC, ×10^9^	6.85 (5.82; 8.03)	6.69 (5.52; 7.84)	6.90 (6.30; 9.10)
Lymphocytes, ×10^9^	2.03(1.50; 2.40)	2.17 (1.81; 2.39)	1.54 (1.20; 2.41)
Monocytes, ×10^9^	0.55 (0.42; 0.66)*	0.44 (0.37; 0.52)*	0.61 (0.48; 0.64)
Neutrophils, ×10^9^	3.72 (3.36; 4.69)	3.73 (2.89; 4.44)	4.10 (3.71; 4.53)
Eosinophils, ×10^9^	0.10 (0.07; 0.17)	0.15 (0.08; 0.20)	0.16 (0.12; 0.28)
Basophils, ×10^9^	0.07 (0.05; 0.10)	0.07 (0.05; 0.10)	0.11 (0.09; 0.15)

*p < 0.01, severe calcified aortic stenosis (SAS) vs. moderate aortic stenosis (MAS) comparison.

^p < 0.01, patient group vs. healthy volunteers (HV) comparison.

In the comparison group, 30 apparently healthy volunteers (HVs) were enrolled. Due to a limited number of subjects older than 60 lacking signs of any chronic disorder, subjects older than 49 were used as one of the inclusion criteria. Therefore, patients and HVs were significantly age mismatched.

The clinical research protocol was approved by the local Ethics Committee of the Almazov National Medical Research Centre (protocol #83, May 16, 2016) and complies with the Declaration of Helsinki. All patients provided written informed consent.

### Histological and Immunohistochemical Analyses of Excised Aortic Valves

Excised aortic valves from the patients with severe aortic stenosis were collected after surgical heart valve replacement. Tissue samples were fixed in 10% buffered formalin and embedded in paraffin after the decalcination. Sections (2 μm thick) were stained with hematoxylin and eosin, and by van Gieson. Specimens were examined with computer-assisted morphometric analysis using the Leica LAS Image Analysis System (Leica QWin Plus v3, Leica Microsystems Imaging Solutions Ltd, Cambridge, UK). The amount of calcium within each sample was assessed at 200× magnification and calculated at 10 fields of view by a single investigation.

According to the amount of calcium measured by pathomorphological slides, all the samples were rated using the following scale: 1—mild calcification, 2—moderate calcification, 3—strongly pronounced (subtotal) calcification, and 4—total calcification. Thus, the pathomorphology study of excised heart valves showed that 4 patients had moderate, 24 had subtotal, and 10 had total calcinosis in heart cusps containing calcium deposits.

Aortic valve infiltration of inflammatory cells was examined immunohistochemically using polyclonal anti-human antibodies to CD3 (Dako Denmark A/S, Glostrup, Denmark) and monoclonal anti-human antibodies to CD39 (BioLegend, Inc., USA) and to CD73 (BioLegend, Inc., USA). Cell count was performed separately for CD3-, CD39-, and CD73-positive cells at 200× magnification and calculated as a mean cell count per 1 mm^2^ at 10 fields of view.

### Blood Sample Collection and Preparation

Cubital vein samples from patients and healthy donors were collected into test tubes added with K_3_EDTA. Clinical blood analysis was performed by using Cell-DYN Ruby Hematology Analyzer (Abbot, USA).

The relative distribution and percentage of T-cell subsets expressing CD39 and CD73 were evaluated by flow cytometry and previously described (Nurkhametova et al., 2018). In brief, 100 μl of peripheral blood was stained with the following anti-human antibody cocktail: CD39-FITC (clone A1, cat. 328206, BioLegend, Inc., USA), CD25-PE (clone B1.49.9, cat. A07774, Beckman Coulter, USA), CD62L-ECD (clone DREG56, cat. IM2713U, Beckman Coulter, USA), CD45R0-PC5.5 (clone UCHL1, cat. IM2712U, Beckman Coulter, USA), CD4-PC7 (clone SFCI12T4D11 (T4), cat. 737660, Beckman Coulter, USA), CD8-APC (clone B9.11, cat. IM2469, Beckman Coulter, USA), CD3-APC-Alexa Fluor 750 (clone UCHT1, cat. A94680, Beckman Coulter, USA), CD73-Pacific Blue (clone AD2, cat. 344012, BioLegend, Inc., USA), and CD45-Krome Orange (clone J33, cat. A96416, Beckman Coulter, USA). Staining protocols were performed in accordance with the manufacturer’s recommendations. Optimal combinations of antibodies directly conjugated with various fluorochromes were used according to Mahnke et al. (Mahnke and Roederer, 2007). Samples were stained with antibodies in the dark, at room temperature, for 15 min. After that, red blood cells were lysed by adding 975 μl of VersaLyse Lysing Solution (cat. A09777, Beckman Coulter, USA) supplied with 25 μl of IOTest 3 Fixative Solution (cat. A07800, Beckman Coulter, USA) in the dark, at room temperature, for 15 min. Then, all samples were washed once with PBS for 7 min by centrifuging at 330*g*. Next, cells were resuspended in 250 μl of PBS supplied with 2% neutral formalin (cat. HT5011-1CS, Sigma-Aldrich Co., USA) and subjected to flow cytometry analysis by using Navios 3/10 flow cytometer equipped with 405-, 488-, and 638-nm diode lasers (Beckman Coulter, USA).

### Flow Cytometry Analysis

**Figure 1:**
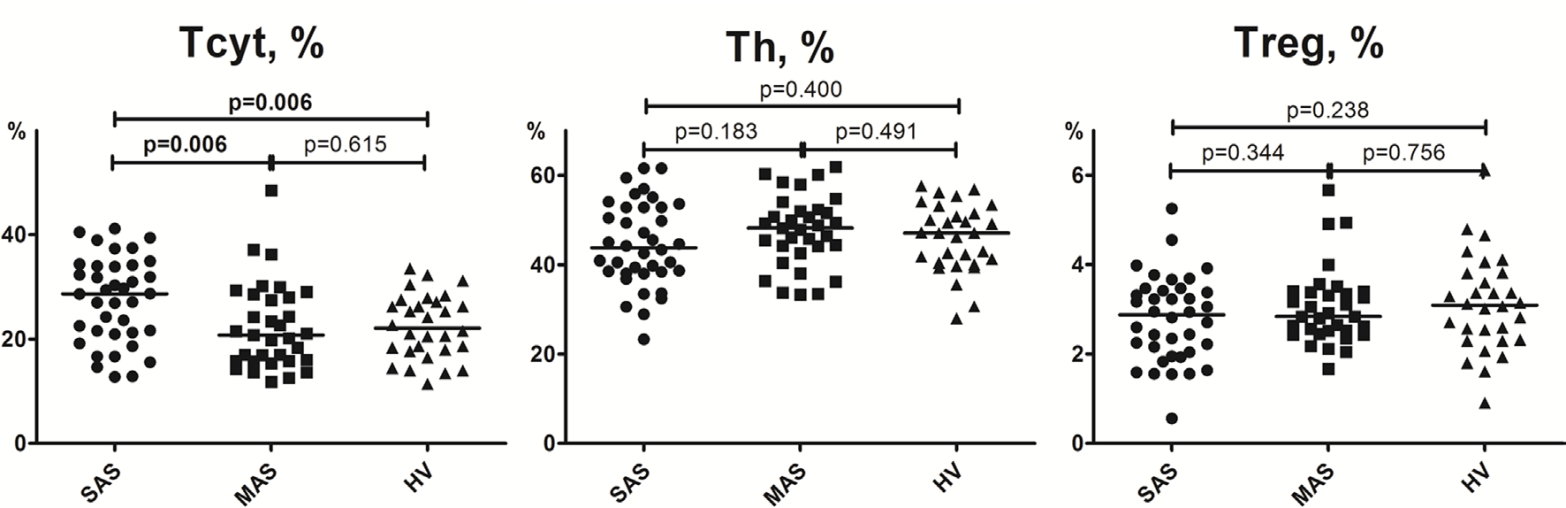
Percentage of CD3+CD4+, CD3+CD8+, and CD3+CD4+CD25hi T-cell subsets in peripheral blood. Note. SAS (black circles, *n* = 38), severe aortic stenosis; MAS (black squares, *n* = 33), moderate aortic stenosis; HV (black triangles, *n* = 30), healthy volunteers. Differences between groups were evaluated by using nonparametric Mann–Whitney *U*-test.

Gating strategy used for evaluating T-cell subset distribution is presented in **Figure 1** (Supplementary Information). T helper (Th) and cytotoxic T-cell subsets (Tcyt) were identified by using mouse anti-human CD3, CD4, and CD8 antibodies, as follows: Th cells were defined as CD3+CD4+ T cells; Tcyt, as CD3+CD8+ T cells. Regulatory T cells (Tregs) were characterized by expression of CD3, CD4, and high IL-2R alpha chain (CD25high) levels (Garcia Santana et al., 2014).

Further, markers CD45R0 and CD62L were used to assess differentiation stage of Th, Tcyt, and Treg subsets. In particular, by co-staining with protein tyrosine phosphatase short isoform CD45R0 and adhesion molecule L-selectin (CD62L), T cells can be subdivided into naïve (naïve, CD45R0−CD62L+), central memory (CM, CD45R0+CD62L+), effector memory (EM, CD45R0+CD62L−), and terminally differentiated CD45RA-positive effector T cells (TEMRA, CD45R0−CD62L−). Finally, the expression of CD39 and CD73 was evaluated in all T-cell subsets mentioned above. Gaiting strategy (**Supplementary Figure 1**) applied in the study was described previously in detail (Golovkin et al., 2017; Nurkhametova et al., 2018).

### Examination of Purinergic Signaling Enzymes in Interstitial Cells Derived from Calcified Aortic Valves

Patients with severe aortic stenosis underwent surgical heart valve replacement. Primary interstitial cells were obtained from the leaflets of the excised heart valves, isolated by using collagenase, and cultured until confluent monolayer as it is previously described (Kostina et al., 2018; Malashicheva et al., 2018). Cells obtained after passages 2–4 were used in further experiments. For this, 12-well plates were seeded with 160,000 cells/well followed by incubating with adenosine (30 mmol) or ATP (100 mmol) 24 h later according to Mahmut et al. (Mahmut et al., 2015). Control cells were unstimulated. Cell phenotyping was performed on days 1, 3, and 7.

Cell staining with anti-human CD39-FITC (clone A1, cat. 328206, BioLegend, Inc., USA) and CD73-PE (clone AD2, cat. 550257, BD Pharmingen™, USA) was performed in accordance with the manufacturer’s recommendations followed by collecting the data on flow cytometer Guava EasyCyte 8 (Millipore, USA).

Mean intensity fluorescence (MIF) for CD39- and CD73-positive staining was analyzed by comparing it with the intensity of isotype-match control antibodies conjugated with relevant fluorochrome followed by estimating the percentage of T-cell subsets expressing CD39−CD73−, CD39−CD73+, CD39+CD73+, and CD39−CD73−.

### qPCR

RNA from cultured cells was isolated using Extract RNA (Eurogene, Russia). Total RNA (1 μg) was reverse transcribed with MMLV RT kit (Eurogen, Russia). Real-time PCR was performed with 1 μl of cDNA and SYBR Green PCR Master Mix (Eurogen, Russia) in the LightCycler system using specific forward and reverse primers for target genes. The corresponding gene expression level was normalized to *GAPDH* from the same samples. Changes in target gene expression levels were calculated as fold differences using the comparative ΔΔCT method (F: AATGAAGGGGTCATTGATGG; R: AAGGTGAAGGTCGGAGTCAA) (Kostina et al., 2018; Malashicheva et al., 2018). *RUNX2* (runt-related transcription factor 2), (F:TGGATCACCTGAAAATGCTG;R:CGAAATCCCAACTCCGATA) *OPN* (osteopontin), (F: TCACCTGTGCCATACCAGTTAAA; R:TGGGTATTTGTTGTAAAGCTGCTT) and *BMP2* (bone morphogenetic protein 2) (F: GCCAGCCGAGCCAACAC; R: CCCACTCGTTTCTGGTAGTTCTTC) were investigated on the seventh day after ATP or adenosine stimulation.

### Statistical Analysis

The data were analyzed by using Navios Software v.1.2 and Kaluza™ software v.2.0 (Beckman Coulter, USA). Statistical analysis was performed by using Statistica 8.0 (StatSoft, USA) and GraphPad Prism 4.00 for Windows (GraphPad Prism Software Inc., USA) software. Normality was checked by using Pearson’s chi-squared test. The data were presented as a percentage of positive cells out of total T-cell subset population as well as absolute number per 1 µL of peripheral blood shown as median ± interquartile range Me (25%; 75%). A dispersion analysis was carried out by using ANOVA. Significance was assessed by using a non-parametric Mann–Whitney *U*-test as well as Student’s *t*-test. Correlation analysis was performed using Spearman rank test. Significance was set at *p* < 0.05.

Multivariate comparison was done by applying a discriminant analysis by using Statistica 8.0 (StatSoft, USA) software. A stepwise analysis enumerating steps, *p*-value significance level, and *F*-test were applied. A discrimination level was evaluated by assessing Wilks’ lambda. Significance of identifying criterion was determined after drawing scatterplots for canonical values and calculating classification value as well as the Mahalanobis squared distance. Statistical discriminant analysis procedure was described previously in detail (Barbarash et al., 2016; Ignatieva et al., 2017).

Discriminant analysis was used to determine which parameters in peripheral T subsets and their surface CD39 and CD73 expression might allow to subdivide patients into various groups, and whether they represent true separate groups. Also, discriminant analysis allows to identify the most common parameters for each group.

## Results

### Clinical Description of Patients

Clinical parameters of subjects enrolled to the study are shown in **Table 1**.

Significant differences (*p* < 0.01) were found in age of patients and control persons due to objective issues related to enrollment of apparently HVs older than 60 bearing comorbidities or conditions satisfying the exclusion criteria. However, studies aimed at investigating purinergic receptors expressed on various T-cell subsets revealed that prominent differences were found only in patients younger and older than 45 (not older than 60) (Fang et al., 2016). It allowed to perform further comparison with the control group detailed above by neglecting significant age-related difference between groups.

Hematological parameters (WBC total, count of lymphocytes, neutrophils, and eosinophils) did not significantly differ between SAS patients and healthy donors. However, monocyte counts were significantly decreased in MAS patients compared with HVs as well as in SAS vs. MAS patients (**Table 1**).

### Major T-Cell Subsets Found in Peripheral Blood

**Figure 2:**
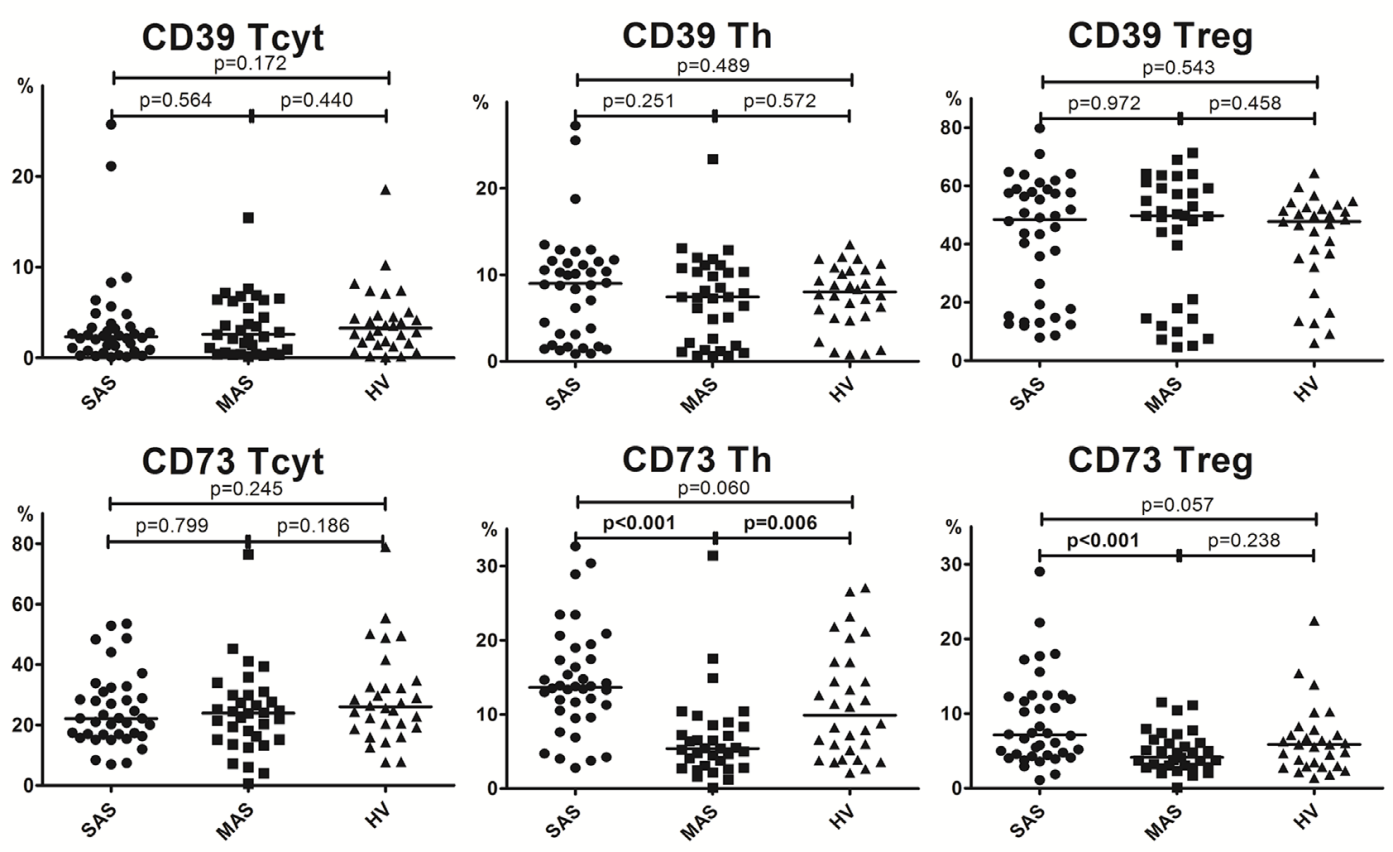
Percentage of major Th, Tcyt, and Treg subsets (gating strategy based on CD45R0 and CD62L expression). Note. SAS (black circles, *n* = 38), severe aortic stenosis; MAS (black squares, *n* = 33), moderate aortic stenosis; HV (black triangles, *n* = 30), healthy volunteers. Naïve (CD45R0−CD62L+); CM, central memory (CD45R0+CD62L+); EM, effector memory (CD45R0+CD62L−); TEMRA, terminally differentiated CD45RA-positive effector memory (CD45R0−CD62L−); Treg, regulatory T cells. Differences between groups were evaluated by using nonparametric Mann–Whitney *U*-test.

While examining T cells, it was found that MAS and SAS patients differed in terms of relative distribution of cytotoxic T-cells and the absolute number of regulatory T cells (**Figures 1** and **2**, **Supplementary Table 1**). Moreover, the absolute number of cytotoxic T cells and TEMRA subset was significantly higher in SAS vs. MAS patients and HVs. However, the absolute and relative number of naïve T helper cells and the absolute number of regulatory T cells were significantly higher in MAS vs. SAS patients (**Supplementary Table 1**). In addition, the relative number of naïve Tregs was significantly (*p* < 0.01) decreased in SAS patients.

Importantly, it is worth noting that the level of all naïve T-cell (i.e., cytotoxic, helper, and regulatory) subsets was lowered in patients with severe vs. moderate aortic stenosis (**Figure 2**).

### Expression of CD73 and CD39 by Major T-Cell Subsets

**Figure 3:**
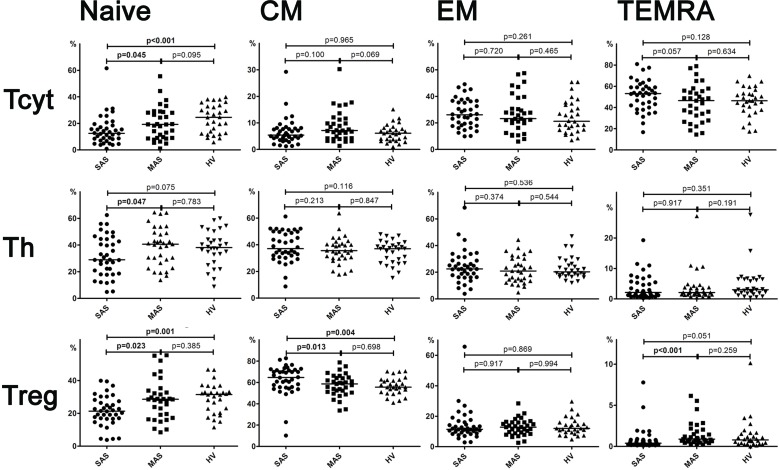
Percentage of CD39 and CD73 expression by Th, Tcyt, and Treg subsets in peripheral blood. Note. SAS (black circles, *n* = 38), severe aortic stenosis; MAS (black squares, *n* = 33), moderate aortic stenosis; HV (black triangles, *n* = 30), healthy volunteers. Differences between groups were evaluated by using nonparametric Mann–Whitney *U*-test.

Analyzing the exonuclease expression on dominant peripheral T-cell subsets revealed no significant differences in the percentage of CD39-positive T cells between groups (**Figure 3**). However, MAS patients vs. HVs were noted to contain significantly lower percentage of Th cells expressing CD73 (*p* = 0.006), whereas SAS patients vs. HVs tended to contain higher percentage of this subset (*p* = 0.06). Comparing SAS vs. MAS patients demonstrated that the percentage of CD73+ Th cells was significantly elevated (*p* < 0.001). Finally, Treg cells were also noted to contain higher percentage of CD73-positive subset in SAS vs. MAS patients.

### Subsets of Peripheral Blood Cytotoxic T Cells, Th, and Tregs

A detailed analysis of T-cell differentiation stages revealed that number of naïve (CD45R0−CD62L+) T cells in all three populations, compared with the HV group, was profoundly decreased in SAS vs. MAS patients as well as in cytotoxic and regulatory T-cell subsets (**Figure 2**). The absolute number of naïve Th and Treg cells was also significantly lower in SAS vs. MAS patients (*p* < 0.05 and *p* < 0.01, respectively). However, on naïve Tcyt cells, this parameter did not differ between groups. In contrast, significantly (*p* = 0.02) increased the percentage of TEMRA (CD45R0−CD62L−) CD8+ T cells was noted in SAS patients vs. HV group. Moreover, MAS vs. SAS patients contained significantly higher percentage and absolute number of this regulatory T-cell subset (*p* < 0.01).

By analyzing various central memory (СМ, CD45R0+CD62L+), T-cell subsets revealed that the absolute number of cytotoxic T cells was significantly increased in MAS patients vs. HV group, whereas the percentage of Tregs within this population was increased in SAS vs. MAS patients and HV group.

No significant differences were observed in any EM T-cell subsets among groups.

### Expression of Ectonucleotidases CD39 and CD73 by Different Subsets of Th, Tcyt, and Tregs

#### Expression of CD73 and CD39 by T Helper Subsets

**Figure 4:**
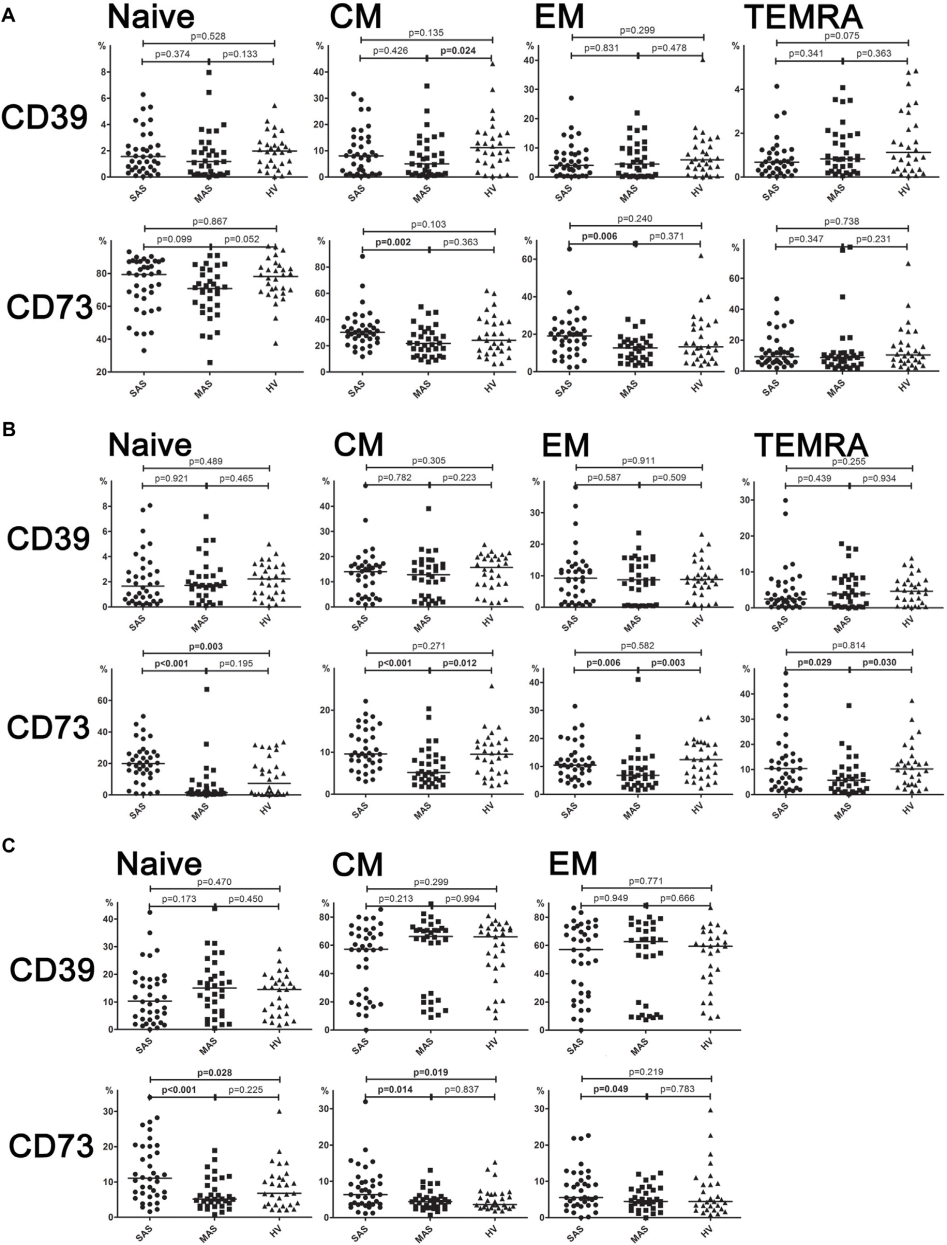
**(A)** Expression of ectonucleotidases CD39 and CD73 by different subsets of cytotoxic T-lymphocytes. Differences between groups were evaluated by using nonparametric Mann–Whitney U-test. **(B)** Expression of ectonucleotidases CD39 and CD73 by various T helper cell subsets. Differences between groups were evaluated by using nonparametric Mann–Whitney U-test. **(C)** Expression of ectonucleotidases CD39 and CD73 by various Treg cell subsets. Differences between groups were evaluated by using nonparametric Mann–Whitney *U*-test.

It was shown that CD73 expression was significantly higher in SAS vs. MAS patients (**Figure 4A**) noted in all EM, CM, TEMRA, and naïve Th cell subsets. However, only the latter were significantly increased (*p* = 0.003) in patients compared with HVs. Surprisingly, the percentage of CD73 Th cell subsets differed to a greater extent in MAS patients vs. HV group, whereas it was decreased in EM (*p* = 0.003), CM (*p* = 0.012), and TEMRA (*p* = 0.030) cells.

#### Expression of CD73 and CD39 by Cytotoxic T-Cell Subsets

Despite the fact that total level of CD39 and CD73 expressed on cytotoxic T cells did not differ, it was found that the percentage of CD39+ CM Tcyt cells was significantly decreased in MAS patients vs. HVs (*p* = 0.024, **Figure 4B**). Moreover, SAS vs. MAS patients were noted to have significantly higher percentage of CD73+ EM Tcyt (*p* = 0.006) and CD73+ CM Tcyt (*p* = 0.002). However, no significant differences were found in the percentage of cytotoxic T-cell subsets expressing either CD39 or CD73 as compared with those of HVs.

#### Expression of CD73 and CD39 on Regulatory T-Cell Subsets

Regarding level of expression for CD39 on Тregs, no significant differences were observed between both patient groups or when compared with HV (**Figure 4C**). However, the expression of CD73 in patients significantly differed in all three populations such as EM (*p* = 0.049), CM (*p* = 0.044), and naïve (*p* < 0.001) T cells. Upon that, it was significantly higher in SAS patients vs. HVs on CM Treg and naïve Treg cells.

### Correlations of T-Cell Subsets and Severity of Calcification

All excised aortic valves from patients with SAS were examined by pathomorphological counting of the amount of calcium in each sample. From the 38 explanted valves, 4 had moderate, 24 had subtotal, and 10 had total calcinosis. In MAS patients, a multislice spiral computed tomography with Agatston calcium scoring was performed to identify the calcification process. These gave us the opportunity to perform the correlation analysis between T-cell subsets and severity of calcification. Significant results for SAS and MAS patients are presented in **Tables 2** and **3**.

**Table 2:** Significant correlations between T-cell subsets and severity of calcification in SAS patients.

T-cell subsets	*r *(*p* < 0.05)
Naïve Tregs, abs	−0.374
Tregs	0.388
CM Tregs	0.417
Naïve Tregs	−0.345
CD39 CM Tcyt	0.438

**Table 3:** Significant correlations between T-cell subsets and severity of calcification in MAS patients according to multislice spiral computed tomography with Agatston calcium scoring.

T-cell subsets	*r *(*p* < 0.05)
Th	0.429
CD4+CD8dim	0.513
CD4+CD8dim, abs	0.474
CD8+CD4dim	0.376
TEMRA Th	−0.429
CD73 EM Th	0.385
CD73 naïve Tregs	0.379
CD73 CM Tregs	0.418

It is noteworthy that there were no common T-cell subsets that could demonstrate the correlations for SAS and MAS patients. For SAS patients, interrelations were observed basically for Tregs and their subsets. Meanwhile, for MAS patients, there were significant positive correlations for CD4+CD8dim and CD8+CD4dim subsets. Besides CD73 expression in EM Th, naïve Tregs and CM Tregs demonstrated their potential interconnection with aortic valve calcification process.

### Discriminant Analysis

**Figure 5:**
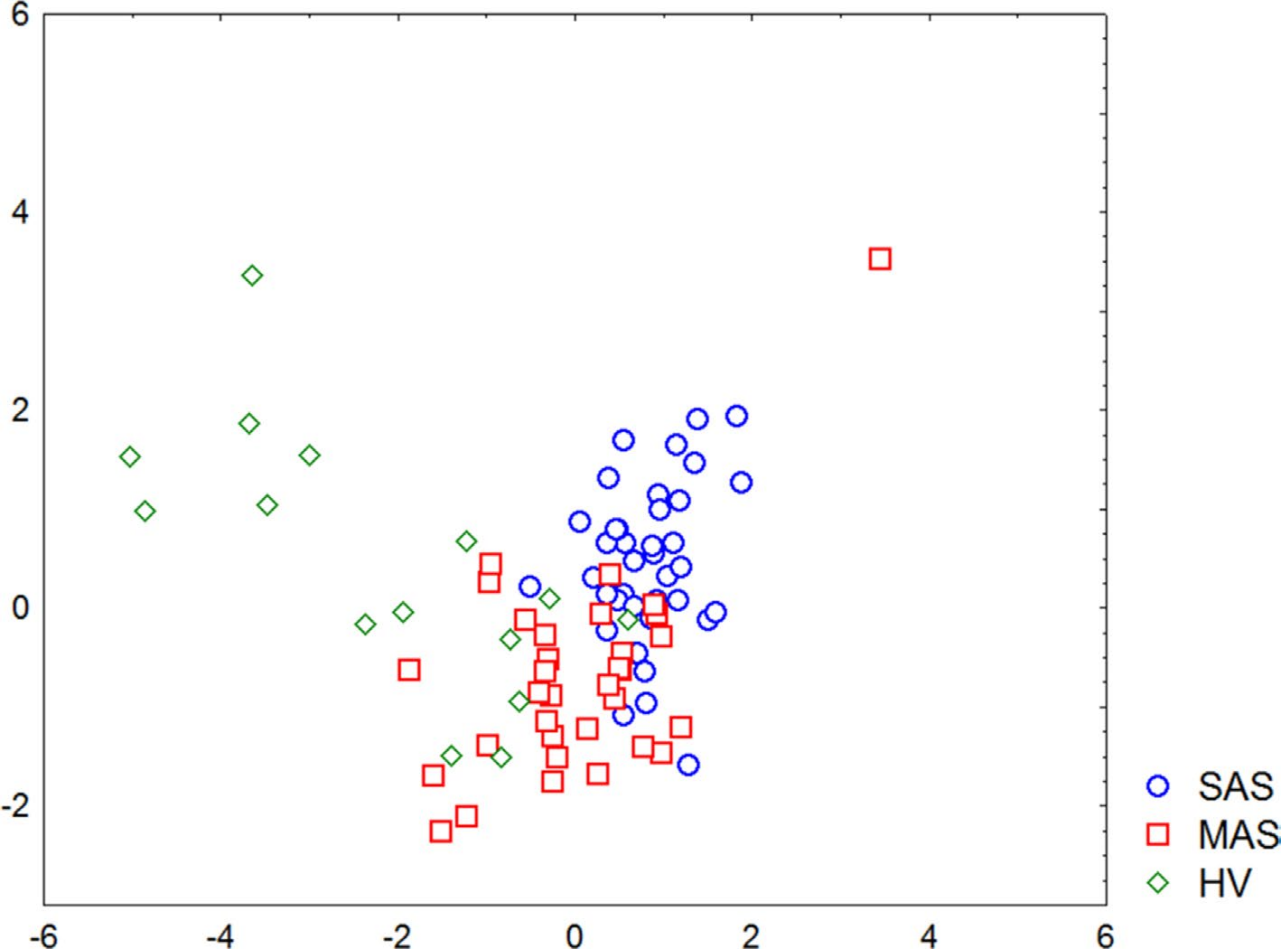
Graphic distribution of SAS and MAS patients as well as healthy volunteers analyzed by discriminant analysis.

A discriminant analysis carried out by using a forward stepwise model consisting of 10 steps (total 45 variables) demonstrated that the top significance was documented while assessing the percentage of CD73−CD39− T cells, CD3+ naïve Tregs, CD73+CD39− T cells, CD73−CD39+ T cells, and TEMRA Tcyt as well as counting the absolute numbers of CD8+CD4dim and TEMRA Tregs (**Table 4**). Partition of the examined groups based on the results from the discriminant analysis is depicted in **Figure 5**.

**Table 4:** Peripheral T-cell subset composition and surface expression of purinergic enzymes in SAS and MAS patients vs. HV group assessed by discriminant analysis.

Parameter	Wilks’ lambda	*F*-test (2.74)	*p*-level
CD73−CD39− T cells	0.613	27.93	<0.001
CD3+	0.524	18.58	<0.001
Naïve Tregs	0.420	7.52	<0.001
CD8+CD4dim, abs	0.384	3.68	0.029
CD73+CD39− T cells	0.465	12.33	<0.001
CD73-CD39+ T cells	0.450	10.75	<0.001
TEMRA Tregs, abs	0.386	3.92	0.024
TEMRA Tcyt	0.381	3.37	0.039
CM Th	0.364	1.63	0.202
CD4+CD8dim, abs	0.363	1.45	0.238

number of steps = 10; Wilks’ lambda = 0.34939; F(20,148) = 5.1192; p < 0.00001.

### Morphological Analysis of Excised Aortic Valves from SAS Patients

**Figure 6:**
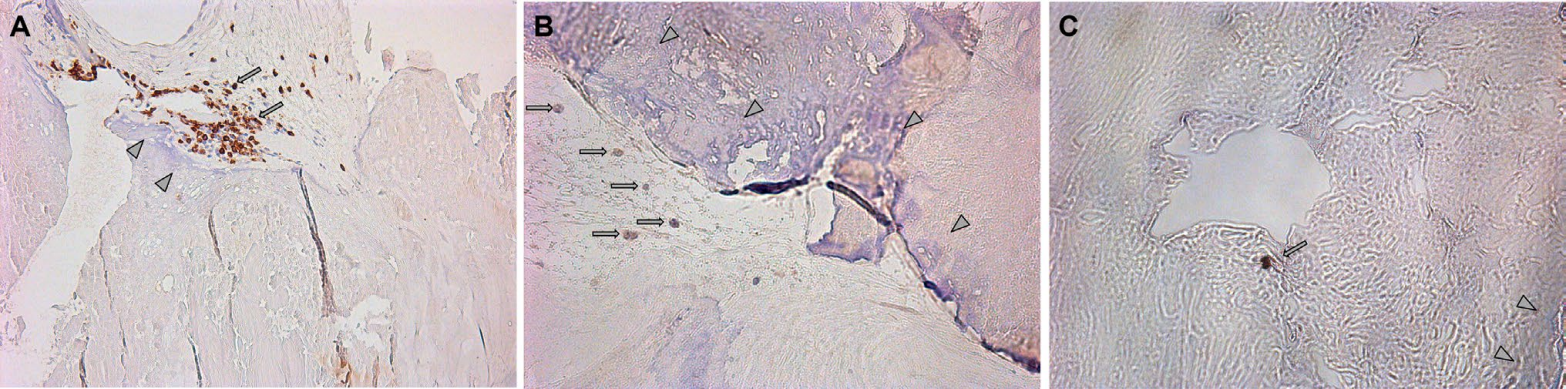
**(A)** Visualization of CD3-positive cells in the excised aortic valves. Focuses of calcification are shown by arrowheads; CD3-positive (brown) cells are shown by arrows. **(B)** Visualization of CD39-positive cells in the excised aortic valves. Focuses of calcification are shown by arrowheads; CD39-positive (brown) cells are shown by arrows. **(C)** Visualization of CD73-positive cells in the excised aortic valves. Focuses of calcification are shown by arrowheads; CD73-positive (brown) cells are shown by arrows.

Leaflets of excised aortic valves were irregularly infiltrated by the CD3-positive cells. Their median amount in all samples was 16.5 (12; 48) cells per mm^2^ (**Figure 6A**). CD39-positive cells were rare. Many fields of view were free of them. Meanwhile, groups of positive cells were visualized on some fields (**Figure 6B**). The CD39+/CD3+ ratio was approximately 1/17. CD73-positive cells were found only on limited fields of view as single rare objects (**Figure 6C**). CD3+, CD39+, and CD73+ cells were presented basically in the calcified regions of the aortic valve tissues.

### Aortic Valve-Derived Interstitial Cells

#### Baseline Expression of CD39 and CD73 on Aortic Valve-Derived Interstitial Cells

We found that the primary culture of interstitial cells derived from calcified aortic valves was mainly presented by CD39−CD73− [93.95 (92.22; 96.44)%] cells. At the same time, 5.05 (3.10; 5.58)% of cells exhibited the CD39−CD73+ phenotype, whereas 0.66 (0.24; 1.76)% of them were CD39+CD73+. Moreover, further cell passages were found to increase their proportion up to 99.09 (98.52; 99.61)%, whereas for CD39−CD73+, their proportion was down to 0.53 (0.36; 0.79)%. Moreover, the proportion of CD39−CD73− increased even further, comprising up to 99.09 (98.52; 99.61)% at later passages, whereas for CD39−CD73+, it decreased down to 0.53 (0.36; 0.79)%.

Assessing level of surface expression for CD39 and CD73 revealed no significant differences when aortic valve-derived interstitial cells were stimulated with ATP or adenosine for 24 h. However, a 7-day incubation with ATP or adenosine resulted in significantly increased percentage of CD39−CD73+ cells from 4.78 (3.08; 6.36) to 12.04 (6.68; 18.50)% and to 12.31 (8.82; 15.97)%, respectively (**Supplementary Figure 2A, 2B**), which was due to a relatively decreased proportion of CD39−CD73− population. Finally, no significant changes were observed in the percentage of CD39+CD73+ or CD39+ cells.

#### Purines Addition do not Stimulate Proosteogenic Differentiation of Aortic Valve-Derived Interstitial Cells

We investigated basic osteogenic markers to find out if proosteogenic differentiation of interstitial aortic valve cells has been launched. A 7-day incubation with ATP or adenosine do not reveal the significant increase comparing with unstimulated interstitial cells derived from calcified aortic valves of *RUNX2* (*p* = 0.814 and *p* = 0.720, respectively), *OPN* (*p* = 0.315 and *p* = 0.528, respectively), and *BMP2* (*p* = 0.541 and *p* = 0.603, respectively) gene expression.

Thus, we demonstrated that purine addition influences the ectonucleotidase expression but does not stimulate proosteogenic differentiation.

## Discussion

T cells represent a relatively small cell population infiltrating various tissues. It is known that T cells exhibit an important regulatory effect on inflammatory and reparative processes by releasing signaling cues for macrophages or fibroblasts (Saxena et al., 2014; Hofmann and Frantz, 2015).

CD3+CD4+ and CD3+CD8+ subsets of peripheral blood T cells exhibiting helper and cytotoxic functions, respectively, can be characterized by combining different cell-surface markers, e.g., CD45R0 and CD62L depending on their migration and functional potential. These T-cell subsets are defined as naïve cells (N, CD45R0−CD62L+), central memory cells (CM, CD45R0+CD62L+), effector memory cells (EM, CD45R0+CD62L−), and “terminally differentiated” effector memory cells re-expressing CD45RA antigen (TEMRA, CD45R0−CD62L−). This classification is typically used for CD8+ and CD4+ T cells, although the equivalent TEMRA Th cell subset in healthy donors has not yet been clearly identified due to their low frequency in normal peripheral blood.

After positive and negative selection, naïve CD4+ and CD8+ T cells exit from the thymus into the peripheral blood and express CCR7 and CD62L required for their extravasation through the high endothelial venules into the secondary lymphoid organs, while the presence of CD45RA and lack of CD45R0 indicates that these cell were not encountered by a specific antigen presented by antigen-presenting cells (Sallusto, 2016). Naïve T cells are characterized by highly diverse TCR repertoire encompassing up to 100 million different specificities (Qi et al., 2014), indicating that naïve T cells can respond to various antigens (Kumar et al., 2018). Moreover, in the absence of specific antigen, they also display a high self-renewal potential, but upon specific stimulation in lymphoid tissues, they undergo robust cell expansion followed by differentiation to effector and memory T-cell subsets.

Two major functional T-cell subsets were distinguished (Mahnke et al., 2013) within the CD45RA−CD45R0+ memory T-cell pool: CM T cells, which express lymph node homing molecules CCR7 and CD62L, had very limited effector functions; and CCR7−CD62L− EM T cells are able to migrate to peripheral tissues and exhibit effector functions. Furthermore, CM cells represent a pool of less-differentiated long-living memory cells that recirculate through the secondary lymphoid organs and display high proliferative and renewal capacity. In contrast, EM T cells represent a pool of more-differentiated circulating effector cells that can rapidly enter inflamed tissues due to the upregulated expression of tissue-homing chemokine receptors and adhesion molecules and provide a rapid and effective defense response (Sallusto, 2016). Both CM CD4+ and CD8+ T cells produce high amounts of IL-2 but low levels of other effector cytokines (e.g., IL-4, IL-5, and IFN-γ), whereas EM Th and Tcyt subsets produce a high amount of effector cytokines but a low level of IL-2 (Mahnke et al., 2013).

Compared with naïve and memory T cells, short-lived effector or TEMRA cells display a limited TCR diversity. However, TEMRA cells rapidly migrate to various anatomical sites to promote pathogen clearance by producing inflammatory cytokines and cytotoxicity, yet they exert little proliferative potential antigen-specific stimulation *in vitro*.

We demonstrated that the percentage of peripheral blood Tcyt was insignificantly increased mainly due to TEMRA Tcyt subset (**Supplementary Table 1**), whereas frequency of naïve Tcyt and naïve Treg subsets was decreased in patients in SAS patients vs. HVs, respectively (**Figure 2**). In contrast, in MAS patients vs. HV, the percentage of CM Tcyt subset was significantly elevated (*p* = 0.01).

Despite few significant differences found in T-cell subset profile observed between patients with moderate aortic stenosis and HVs, it is worth noting about multiple differences between SAS and MAS patients mainly due to opposite changes in similar parameters found in HV group.

It was found that the amount of total Tcyt cells, particularly the relative and absolute numbers of TEMRA Tcyt subset in SAS vs. MAS patients, was considerably increased, which could point most likely at their continuous antigen-specific activation, differentiation, and pro-inflammatory activity. Importantly, no such changes in MAS patients vs. HVs were found.

Mazur et al. performed correlation analyses that demonstrated that mean transvalvular pressure gradient positively correlated with the CD4+CD8+ lymphocyte count and fraction (Mazur et al., 2018). Taking into account the great interest on double-positive T-cell population, we also involved them into the analysis, but having the opportunity to identify this population as heterogeneous, we analyzed it separately as CD4+CD8dim and CD8+CD4dim (**Supplementary Table 1**). There were no significant changes in absolute and relative count of these populations between MAS and SAS patients and HVs. Meanwhile, positive correlations of CD4+CD8dim and CD8+CD4dim with Agatston calcium score for MAS patients demonstrated their potential interconnection with aortic valve calcification process.

Previously, it was demonstrated that purinergic signaling played an important role in regulating T-cell functions (Ledderose et al., 2016; Golovkin et al., 2018). T cells migrating into the damaged heart tissues upregulate enzymatic machinery for degrading extracellular ATP and NAD to adenosine (Borg et al., 2017).

Numerous studies support the concept that CD39 controls the rate-limiting step in the degradation of extracellular ATP (Eltzschig et al., 2012). While there are multiple AMP sources, the critical bottleneck for the formation of adenosine is CD73 also undergoing upregulated expression (Bonner et al., 2012). Meanwhile, lack of CD73 on T cells enhances tissue fibrosis (Borg et al., 2017). T cells lacking CD73 show accelerated production of pro-inflammatory and profibrotic cytokines (IL-2, IFN-γ, and IL-17) (Borg et al., 2017) presumably accounting for down-modulated CD73 expression on Th and Tcyt subsets in MAS vs. SAS patients vs. HVs.

There are different cellular sources for AMP formation: monocytes and fibroblasts—both largely devoid of CD73—express CD39 and can only degrade ATP to AMP, which may then reach T cells by diffusion (Bonner et al., 2013; Borg et al., 2017).

CD73-derived adenosine acts in an autocrine manner on adenosine receptors A_2a_R and A_2b_R predominantly expressed by cardiac T cells to counteract pro-inflammatory/antifibrotic cytokines (Borg et al., 2017). For instance, A_2b_R stimulation was reported to suppress the release of TNFα from neutrophils and macrophages (Koeppen et al., 2015), inhibit superoxide anion production in neutrophils (Auchampach et al., 2009), augment anti-inflammatory IL-10 production in macrophages (Nemeth et al., 2005), and promote alternative (M2) macrophage activation (Csoka et al., 2012). In addition, T-cell-derived adenosine might influence cytokine release and differentiation of other cardiac immune cells in a paracrine manner, again involving A_2a_R and A_2b_R (Borg et al., 2017). Meanwhile, CD73 promoting adenosine production is regulated by various cytokines, e.g., interferon (IFN)-β (Kiss et al., 2007), TGFβ, IFN-γ, IL-6, and IL-12 (Regateiro et al., 2011), as well as by paracrine adenosine (Narravula et al., 2000).

While investigating patients with anti-neutrophil cytoplasmic auto-antibody (ANCA)-associated vasculitis (AAV), it was found that on patient T cells, CD39 and CD73 were down-modulated. In CD4+ cells, significant differences in CD73 expression were limited to memory CD45RA− cells, while in CD4+ lymphocytes, differences were significant in both naïve CD45RA+ and memory CD45RA− cells. However, no correlation with disease activity, duration, and ANCA profile was found (Kling et al., 2017).

Previously, it was shown that CD39 and CD73 expression on T cells could be related to the age of human subjects. For instance, CD73 expression on lymphocytes declines with age (Guzman-Flores et al., 2015). Meanwhile, CD39 is more readily induced in CD4 T-cell responses in older individuals (Fang et al., 2016). In our previous investigation with HVs, we found that the number of CD73-expressing naïve T cells was significantly decreased in donors older than 45 compared with younger subjects. However, such pattern was only noted for female subjects (Golovkin et al., 2017). Meanwhile, a significant correlation (*r* = −0.517, *p* < 0.05) between CD73 expression on T-cytotoxic cells and male subjects of older age was shown. Besides, female volunteers demonstrated numerous age-related correlations: naïve Tcyt CD73+ (*r* = −0.520, *p* < 0.05), CM Tcyt CD73+ (*r* = −0.397, *p* < 0.05), naïve Tcyt CD39+ (*r* = 0.361, *p* < 0.05), EM Tcyt CD39+ (*r* = 0.367, *p* < 0.05), and naïve Th CD39+ (*r* = 0.378, *p* < 0.05) (Golovkin et al., 2017). Therefore, CD39 and CD73 expression on T cells may display both age- and sex-related features.

Despite that mean age was lower in HV group, all differences found in our study might not be accounted for only by age-related issues, given especially that the vast majority of them were found between age-matched MAS and SAS patients in terms of T-cell subset composition and percentage of CD39- and CD73-positive cells they contained.

It is known that CD4+CD25+ regulatory T cells (Tregs) are important in maintaining self-tolerance and regulating immune responses in both physiological and pathological settings (Sakaguchi, 2004; Singer et al., 2014; Huang et al., 2015). Tregs demonstrate a great diversity of their composition and functions mediating immune suppression through distinct mechanisms associated with various phenotypical and functional subsets (Caridade et al., 2013). CD39 is a useful marker for identifying CD4+CD25+ Treg cells, even allowing to consider it as a more consistent and reliable marker for Treg cells than CD25 (Deaglio et al., 2007). It was demonstrated that the level of surface CD39 expression on human Tregs parallels that one for intracellular transcription factor FOXP3 (Borsellino et al., 2007; Deaglio et al., 2007). Furthermore, phenotypic analysis comparing CD39+ and CD39− Tregs in adult donors showed that the former expressed a higher level of intracellular FOXP3 and CTLA-4 than did CD39− Tregs (Rissiek et al., 2015). Analyzing chemokine receptor expression revealed that a higher percentage of CD39+ Tregs expressed CCR4, CXCR3, and CCR6 involved in T-cell migration to the site of inflammation, while CCR5 was uniquely expressed by CD39+ Tregs. Upon *in vitro* activation, proteins GARP and LAP anchored to TGFβ were expressed at a higher level on CD39+ Tregs. Furthermore, it was shown that CD39 plays a non-redundant role in mediating suppressive potential of Treg cells. In addition, FOXP3+CD39+ cells efficiently suppressed CD4+CD25− proliferation *in vitro*. Moreover, CD39+ vs. CD39− Treg cells displayed a significantly higher suppressive potential (Deaglio et al., 2007).

In contrast, in our study, CD39 was not expressed by total Treg population. Upon that, no significant differences were revealed comparing aortic stenosis patients vs. HV group. However, it is worth mentioning that low- (<20%) and high-percentage (around 50%) Treg subsets tended to emerge, which was observed in all groups. Earlier, increased CD39 expression on Tregs was shown in HIV patients, which was strongly associated with disease progression (Nikolova et al., 2011). Furthermore, an increase in CD39 expression on Tregs has also been detected in tumors and autoimmune diseases (Bastid et al., 2013). Besides, the increased expression of CD39+ Tregs was associated with a poor prognosis for sepsis patients (Huang et al., 2015).

It has been reported that in humans, only 1–5% of circulating FOXP3+ CD4+ T cells express CD73, while its surface expression on human Tregs can be induced upon activation (Sim et al., 2014). In addition, CD73 on Tregs was confirmed to contribute to its functioning, as its suppression limits their suppressive capacity (Zhao et al., 2017b). Besides its enzymatic function, CD73 could be considered as an adhesion molecule that can regulate cell interaction with extracellular matrix components (Zhao et al., 2017b). It seems that a higher CD73+ level on Tregs, particularly CM and naïve subsets, points at stronger suppressive potential in patients with severe aortic stenosis.

A stepwise discriminant analysis uncovered that T-cell subset composition played a pronounced role in distinguishing various groups. Moreover, the expression of the enzymes metabolizing extracellular ATP were of strongest significance particularly involving T cells exhibiting phenotype CD73−CD39−, CD73+CD39−, and CD73−CD39+. Thus, parameters of T-cell-mediated immunity and purinergic regulation were substantially different in SAS vs. MAS patients as well as in HV group.

Moreover, discriminant analysis uncovered that patients with various stenosis magnitude formed rather separate groups, and major parameters were sufficient for referring each patient to either group. At the same time, the minimum differences between MAS patients and HV group in parameters noted above were found, which was not observed by comparing SAS patients vs. HV group. It seems that this discrepancy was due to the intensity of aortic calcinosis found in both patient groups.

Examining interstitial cells isolated from aortic valve biopsies demonstrated that the majority of them displayed CD39−CD73− phenotype. Upon that, further passages were shown to increase proportion of the cells expressing these markers up to 100%. Besides, our immunohistochemical data demonstrate that despite the presence of CD3-positive cells in the calcified aortic valves tissue, the number of CD39-positive cells is low, and CD73-positive cells are almost absent. Previously, it was shown that the low or even insufficient level of CD39 and CD73 expression was observed on the cells from calcified vs. non-calcified aortic valves (Kaniewska-Bednarczuk et al., 2018), which was accompanied with reduced activity of NTPDase and e5NT by 35% and 50% (Kaniewska-Bednarczuk et al., 2018), respectively, in calcified lesions. In our study, it was noted that stimulation with either ATP or adenosine postponed (for 7 days) upregulated CD73 expression detected on interstitial cells, whereas the percentage of CD39−CD73+ cells was even decreased 24 h after the onset of experiment. Thus, the data obtained in our study confirm a hypothesis (Kaniewska-Bednarczuk et al., 2018) proposing a down-regulated expression and decreased activity of ectoenzymes CD39 and CD73 on the cells from the calcified aortic valves. Despite other data contradicting those of our study, we found higher CD73 expression and activity level for the calcified vs. non-calcified valve cells (Mahmut et al., 2015). Moreover, we also demonstrated that ectonucleotidase expression level did not differ after a short-term stimulation with either adenosine or ATP, perhaps, on the one hand, pointing at the potential role for purinergic signaling in aortic valve calcification and, on the other hand, maybe implying lack of plasticity in enzymatic system under pathological settings that make it unresponsive to the targeted activation. Confirmation of the latter can be considered by the lack of changes in the expression of genes associated with proosteogenic activity.

## Concluding Remarks

Cell functions regulated by purinergic signaling seem to be of importance in various pathological conditions including formation of aortic stenosis and its calcification, which may be related to activity of peripheral blood T-cell subsets and aortic valve interstitial cells. It was found that patients with severe calcified aortic stenosis contained higher percentage of CD73+ Th cells primarily due to CD73+ naïve as well as naïve and CM Treg cell subsets. Along with that, frequency of the same CD73+ T-cell subsets in patients with moderate aortic stenosis was decreased, thereby confirming a concept that the lack or decreased CD73 T-cell expression enhances production of pro-inflammatory and profibrotic cytokines exhibiting overall pro-inflammatory effects. Strikingly, no significant differences in CD39 expression level were found in MAS and SAS patients compared with HV group. Moreover, in our study, a significance of the surface ectonuclease expression on aortic interstitial cells was also demonstrated. In particular, such cells isolated from aortic calcified valve samples turned out to mainly display CD39−CD73− phenotype, which increased percentage of CD73+ cells in response to stimulation with adenosine or ATP. Overall, the data obtained demonstrated that purinergic signaling was involved in the pathogenesis of aortic stenosis and calcification potentially acting *via* various cell types (T cells and aortic interstitial cells), wherein among enzymes, degrading extracellular ATP CD73 rather than CD39 played a prominent role.

## Ethics Statement

The clinical research protocol was approved by the local Ethics Committee of the Almazov Federal Medical Research Centre (protocol #83, May 16, 2016), and complies with the Declaration of Helsinki. All patients provided written informed consent.

## Author Contributions

KI and SM performed flow cytometry research. ZE, MP, TV, and IO created the database of patients and healthy volunteers. MA, SA, and SD performed valve interstitial cells research. GA, KI, and MO designed the research. ML performed all histological and immunohistochemical analyses. GA and KI analyzed the data. GA, KI, and ID wrote the paper.

## Funding

Clinical studies were performed at the Almazov National Medical Research Centre and supported by the Russian Foundation for Basic Research (project 18-015-00016). Laboratory studies were financially supported by the Russian Science Foundation (grant/award no. 19-75-20076).

## Conflict of Interest Statement

The authors declare that the research was conducted in the absence of any commercial or financial relationships that could be construed as a potential conflict of interest.

## References

[B1] Auchampach J. A. Kreckler L. M. Wan T. C. Maas J. E. van der Hoeven D. Gizewski E. (2009). Characterization of the A2B adenosine receptor from mouse, rabbit, and dog. J. Pharmacol. Exp. Ther. 329, 2–13. 10.1124/jpet.108.148270 19141710 PMC2670590

[B2] Barbarash L. Kudryavtsev I. Rutkovskaya N. Golovkin A. (2016). T cell response in patients with implanted biological and mechanical prosthetic heart valves. Mediat. Inflammation 2016, 1937564. 10.1155/2016/1937564 PMC4773556 26989331

[B3] Bastid J. Cottalorda-Regairaz A. Alberici G. Bonnefoy N. Eliaou J.-F. Bensussan A. (2013). ENTPD1/CD39 is a promising therapeutic target in oncology. Oncogene 32, 1743–1751. 10.1038/onc.2012.269 22751118

[B4] Bonner F. Borg N. Burghoff S. Schrader J. (2012). Resident cardiac immune cells and expression of the ectonucleotidase enzymes CD39 and CD73 after ischemic injury. PLoS One 7, e34730. 10.1371/journal.pone.0034730 22514659 PMC3326036

[B5] Bonner F. Borg N. Jacoby C. Temme S. Ding Z. Flogel U. (2013). Ecto-5′-nucleotidase on immune cells protects from adverse cardiac remodeling. Circ. Res. 113, 301–312. 10.1161/CIRCRESAHA.113.300180 23720442

[B6] Bono M. R. Fernández D. Flores-Santibáñez F. Rosemblatt M. Sauma D. (2015). CD73 and CD39 ectonucleotidases in T cell differentiation: beyond immunosuppression. FEBS Lett. 589, 3454–3460. 10.1016/j.febslet.2015.07.027 26226423

[B7] Borg N. Alter C. Görldt N. Jacoby C. Ding Z. Steckel B. (2017). CD73 on T cells orchestrates cardiac wound healing after myocardial infarction by purinergic metabolic reprogramming. Circulation 136, 297–313. 10.1161/CIRCULATIONAHA.116.023365 28432149

[B8] Borsellino G. Kleinewietfeld M. Di Mitri D. Sternjak A. Diamantini A. Giometto R. (2007). Expression of ectonucleotidase CD39 by Foxp3+ Treg cells: hydrolysis of extracellular ATP and immune suppression. Blood 110, 1225–1232. 10.1182/blood-2006-12-064527 17449799

[B9] Caridade M. Graca L. Ribeiro R. M. (2013). Mechanisms underlying CD4+ Treg immune regulation in the adult: from experiments to models. Front. Immunol. 4, 378. 10.3389/fimmu.2013.00378 24302924 PMC3831161

[B10] Csóka B. Himer L. Selmeczy Z. Vizi E. S. Pacher P. Ledent C. (2008). Adenosine A2A receptor activation inhibits T helper 1 and T helper 2 cell development and effector function. FASEB J. 22, 3491–3499. 10.1096/fj.08-107458 18625677 PMC2537430

[B11] Csoka B. Selmeczy Z. Koscso B. Nemeth Z. H. Pacher P. Murray P. J. (2012). Adenosine promotes alternative macrophage activation via A2A and A2B receptors. FASEB J. 26, 376–386. 10.1096/fj.11-190934 21926236 PMC3250237

[B12] Deaglio S. Dwyer K. M. Gao W. Friedman D. Usheva A. Erat A. (2007). Adenosine generation catalyzed by CD39 and CD73 expressed on regulatory T cells mediates immune suppression. J. Exp. Med. 204, 1257–1265. 10.1084/jem.20062512 17502665 PMC2118603

[B13] Dou L. Chen Y. Cowan P. J. Chen X. (2018). Extracellular ATP signaling and clinical relevance. Clin. Immunol. 188, 67–73. 10.1016/j.clim.2017.12.006 29274390

[B14] Eltzschig H. K. Sitkovsky M. V. Robson S. C. (2012). Purinergic signaling during inflammation. N. Engl. J. Med. 367, 2322–2333. 10.1056/NEJMra1205750 23234515 PMC3675791

[B15] Faas M. M. Sáez T. de Vos P. (2017). Extracellular ATP and adenosine: the yin and yang in immune responses? Mol. Aspects Med. 55, 9–19. 10.1016/j.mam.2017.01.002 28093236

[B16] Falk V. Baumgartner H. Bax J. J. De Bonis M. Hamm C. Holm P. J. (2017). ESC/EACTS Guidelines for the management of valvular heart disease. Eur. J. Cardiothorac. Surg. 52 (4), 616–664. 10.1093/ejcts/ezx324 29156023

[B17] Fang F. Yu M. Cavanagh M. M. Hutter Saunders J. Qi Q. Ye Z. (2016). Expression of CD39 on activated T cells impairs their survival in older individuals. Cell Rep. 14, 1218–1231. 10.1016/j.celrep.2016.01.002 26832412 PMC4851554

[B18] Fish R. S. Klootwijk E. Tam F. W. K. Kleta R. Wheeler D. C. Unwin R. J. (2013). ATP and arterial calcification. Eur. J. Clin. Invest. 43, 405–412. 10.1111/eci.12055 23398250

[B19] Garcia Santana C. A. Tung J. W. Gulnik S. (2014). Human Treg cells are characterized by low/negative CD6 expression. Cytom. Part A 85, 901–908. 10.1002/cyto.a.22513 25088497

[B20] Golovkin A. Serebryakova M. Zhiduleva E. Murtazalieva P. Titov V. Irtuga O. (2017). Purinergic signaling receptors expression on peripheral T-lymphocytes of healthy donors. Transl. Med. 4, 46–60. 10.18705/2311-4495-2017-4-5-46-60

[B21] Golovkin A. S. Asadullina I. A. Kudryavtsev I. V. (2018). Purinergic regulation of basic physiological and pathological processes. Med. Immunol. 20, 463–476. 10.15789/1563-0625-2018-4-463-476

[B22] Guzman-Flores J. M. Cortez-Espinosa N. Cortés-Garcia J. D. Vargas-Morales J. M. Cataño-Cañizalez Y. G. Rodríguez-Rivera J. G. (2015). Expression of CD73 and A2A receptors in cells from subjects with obesity and type 2 diabetes mellitus. Immunobiology 220, 976–984. 10.1016/j.imbio.2015.02.007 25770019

[B23] Hofmann U. Frantz S. (2015). Role of lymphocytes in myocardial injury, healing, and remodeling after myocardial infarction. Circ. Res. 116, 354–367. 10.1161/CIRCRESAHA.116.304072 25593279

[B24] Huang H. Xu R. Lin F. Bao C. Wang S. Ji C. (2015). High circulating CD39+ regulatory T cells predict poor survival for sepsis patients. Int. J. Infect. Dis. 30, e57–e63. 10.1016/j.ijid.2014.11.006 25461658

[B25] Ignatieva E. Kostina D. Irtyuga O. Uspensky V. Golovkin A. Gavriliuk N. (2017). Mechanisms of smooth muscle cell differentiation are distinctly altered in thoracic aortic aneurysms associated with bicuspid or tricuspid aortic valves. Front. Physiol. 8, 536. 10.3389/fphys.2017.00536 28790933 PMC5524772

[B26] Kaniewska E. Sielicka A. Sarathchandra P. Pelikant-Małecka I. Olkowicz M. Słomińska E. M. (2014). Immunohistochemical and functional analysis of ectonucleoside triphosphate diphosphohydrolase 1 (CD39) and ecto-5′-nucleotidase (CD73) in pig aortic valves. Nucleosides Nucleotides Nucleic Acids 33, 305–312. 10.1080/15257770.2014.885985 24940684

[B27] Kaniewska-Bednarczuk E. Kutryb-Zajac B. Sarathchandra P. Pelikant-Malecka I. Sielicka A. Piotrowska I. (2018). CD39 and CD73 in the aortic valve—biochemical and immunohistochemical analysis in valve cell populations and its changes in valve mineralization. Cardiovasc. Pathol. 36, 53–63. 10.1016/j.carpath.2018.05.008 30056298

[B28] Kiss J. Yegutkin G. G. Koskinen K. Savunen T. Jalkanen S. Salmi M. (2007). IFN-β protects from vascular leakage via up-regulation of CD73. Eur. J. Immunol. 37, 3334–3338. 10.1002/eji.200737793 18034430

[B29] Kling L. Benck U. Breedijk A. Leikeim L. Heitzmann M. Porubsky S. (2017). Changes in CD73, CD39 and CD26 expression on T-lymphocytes of ANCA-associated vasculitis patients suggest impairment in adenosine generation and turn-over. Sci. Rep. 7, 11683. 10.1038/s41598-017-12011-4 28916770 PMC5601951

[B30] Koeppen M. Harter P. N. Bonney S. Bonney M. Reithel S. Zachskorn C. (2015). Adora2b signaling on bone marrow derived cells dampens myocardial ischemia–reperfusion injury. Anesthesiology 116, 1245–1257. 10.1097/ALN.0b013e318255793c PMC3360806 22531331

[B31] Kostina A. Shishkova A. Ignatieva E. Irtyuga O. Bogdanova M. Levchuk K. (2018). Different Notch signaling in cells from calcified bicuspid and tricuspid aortic valves. J. Mol. Cell. Cardiol. 114, 211–219. 10.1016/j.yjmcc.2017.11.009 29158034

[B32] Kumar B. V. Connors T. J. Farber D. L. (2018). Human T cell development, localization, and function throughout life. Immunity 48, 202–213. 10.1016/j.immuni.2018.01.007 29466753 PMC5826622

[B33] Ledderose C. Bao Y. Kondo Y. Fakhari M. Slubowski C. Zhang J. (2016). Purinergic signaling and the immune response in sepsis: a review. Clin. Ther. 38, 1054–1065. 10.1016/j.clinthera.2016.04.002 27156007 PMC4875817

[B34] Mahmut A. Boulanger M.-C. Bouchareb R. Hadji F. Mathieu P. (2015). Adenosine derived from ecto-nucleotidases in calcific aortic valve disease promotes mineralization through A2a adenosine receptor. Cardiovasc. Res. 106, 109–120. 10.1093/cvr/cvv027 25644539

[B35] Mahnke Y. D. Roederer M. (2007). Optimizing a multi-colour immunophenotyping assay. Clin. Lab. Med. 27, 1–18. 10.1016/j.cll.2007.05.002 17658403 PMC2034273

[B36] Mahnke Y. D. Brodie T. M. Sallusto F., and Roederer M, L. E. (2013). The who’s who of T-cell differentiation: human memory T-cell subsets. Eur. J. Immunol. 43, 2797–2809. 10.1002/eji.201343751 24258910

[B37] Malashicheva A. Irtyuga O. Kostina A. Golovkin A. Gordeev M. Moiseeva O. (2018). Osteogenic potential of adipose mesenchymal stem cells is not correlated with aortic valve calcification. Biol. Commun. 63, 117–122. 10.21638/spbu03.2018.204

[B38] Mazur P. Mielimonka A. Natorska J. Wypasek E. Gawęda B. Sobczyk D. (2018). Lymphocyte and monocyte subpopulations in severe aortic stenosis at the time of surgical intervention. Cardiovasc. Pathol. 35, 1–7. 10.1016/j.carpath.2018.03.004 29727769

[B39] Narravula S. Lennon P. F. Mueller B. U. Colgan S. P. (2000). Regulation of endothelial CD73 by adenosine: paracrine pathway for enhanced endothelial barrier function. J. Immunol. 165, 5262–5268. 10.4049/jimmunol.165.9.5262 11046060

[B40] Nemeth Z. H. Lutz C. S. Csoka B. Deitch E. A. Leibovich S. J. Gause W. C. (2005). Adenosine augments IL-10 production by macrophages through an A2B receptor-mediated posttranscriptional mechanism. J. Immunol. 175, 8260–8270. 10.4049/jimmunol.175.12.8260 16339566 PMC2000336

[B41] Nikolova M. Carriere M. Jenabian M. A. Limou S. Younas M. Kök A. (2011). CD39/adenosine pathway is involved in AIDS progression. PLoS Pathog. 7 (7), e1002110. 10.1371/journal.ppat.1002110 21750674 PMC3131268

[B42] Nurkhametova D. Kudryavtsev I. Khayrutdinova O. Serebryakova M. Altunbaev R. Malm T. (2018). Purinergic profiling of regulatory T-cells in patients with episodic migraine. Front. Cell. Neurosci. 12, 326. 10.3389/fncel.2018.00326 30319363 PMC6167492

[B43] Ohta A. Sitkovsky M. (2001). Role of G-protein-coupled adenosine receptors in downregulation of inflammation and protection from tissue damage. Nature 414, 916–920. 10.1038/414916a 11780065

[B44] Qi Q. Liu Y. Cheng Y. Glanville J. Zhang D. Lee J.-Y. (2014). Diversity and clonal selection in the human T-cell repertoire. Proc. Natl. Acad. Sci. 111, 13139–13144. 10.1073/pnas.1409155111 25157137 PMC4246948

[B45] Regateiro F. S. Howie D. Nolan K. F. Agorogiannis E. I. Greaves D. R. Cobbold S. P. (2011). Generation of anti-inflammatory adenosine by leukocytes is regulated by TGF-beta. Eur. J. Immunol. 41, 2955–2965. 10.1002/eji.201141512 21770045

[B46] Rissiek A. Baumann I. Cuapio A. Mautner A. Kolster M. Arck P. C. (2015). The expression of CD39 on regulatory T cells is genetically driven and further upregulated at sites of inflammation. J. Autoimmun. 58, 12–20. 10.1016/j.jaut.2014.12.007 25640206

[B47] Sakaguchi S. (2004). Naturally arising CD4+ regulatory t cells for immunologic self-tolerance and negative control of immune responses. Annu. Rev. Immunol. 22, 531–562. 10.1146/annurev.immunol.21.120601.141122 15032588

[B48] Sallusto F. (2016). Heterogeneity of human CD4 + T cells against microbes. Annu. Rev. Immunol. 34, 317–334. 10.1146/annurev-immunol-032414-112056 27168241

[B49] Saxena A. Dobaczewski M. Rai V. Haque Z. Chen W. Li N. (2014). Regulatory T cells are recruited in the infarcted mouse myocardium and may modulate fibroblast phenotype and function. AJP Hear. Circ. Physiol. 307, H1233–H1242. 10.1152/ajpheart.00328.2014 PMC4200341 25128167

[B50] Sim G. C. Martin-Orozco N. Jin L. Yang Y. Wu S. Washington E. (2014). IL-2 therapy promotes suppressive ICOS+Treg expansion in melanoma patients. J. Clin. Invest. 124, 99–110. 10.1172/JCI46266 24292706 PMC3871216

[B51] Singer B. D. King L. S. D’Alessio F. R. (2014). Regulatory T cells as immunotherapy. Front. Immunol. 5, 46. 10.3389/fimmu.2014.00046 24575095 PMC3920065

[B52] Stagg J. Smyth M. J. (2010). Extracellular adenosine triphosphate and adenosine in cancer. Oncogene 29, 5346–5358. 10.1038/onc.2010.292 20661219

[B53] Towler D. A. (2017). Commonalities between vasculature and bone. Circulation 135, 320–322. 10.1161/CIRCULATIONAHA.116.022562 28115412 PMC5291142

[B54] Trabanelli S. Očadlíková D. Gulinelli S. Curti A. Salvestrini V. de Paula Vieira R. (2012). Extracellular ATP exerts opposite effects on activated and regulatory CD4+ T cells via purinergic P2 receptor activation. J. Immunol. 189, 1303–1310. 10.4049/jimmunol.1103800 22753942

[B55] Winchester R. Wiesendanger M. O’Brien W. Zhang H.-Z. Maurer M. S. Gillam L. D. (2011). Circulating activated and effector memory T cells are associated with calcification and clonal expansions in bicuspid and tricuspid valves of calcific aortic stenosis. J. Immunol. 187, 1006–1014. 10.4049/jimmunol.1003521 21677140 PMC3131440

[B56] Zhao H. Bo C. Kang Y. Li H. (2017a). What else can CD39 tell us? Front. Immunol. 8, 727. 10.3389/fimmu.2017.00727 28690614 PMC5479880

[B57] Zhao H. Liao X. Kang Y. (2017b). Tregs: where we are and what comes next? Front. Immunol. 8, 1578. 10.3389/fimmu.2017.01578 29225597 PMC5705554

