# Supplementary material for: CD73 Rather Than CD39 Is Mainly Involved in Controlling Purinergic Signaling in Calcified Aortic Valve Disease
Source: Front Genet. 2019 Jul 25;10:604. doi: 10.3389/fgene.2019.00604 (PMC6669234; doi:10.3389/fgene.2019.00604)
Supplement: Supplementary file 1 [file Table_1.docx]

**Supplementary Table 1.** **Main T cell subset composition**

| T cells subsets | Severe stenosis (n=38) | Moderate stenosis (n=33) | Healthy volunteers (n=30) |
| --- | --- | --- | --- |
| Tcyt, abs | 517 (341; 662)* | 424 (281; 563) | 393 (232;482) |
| Naïve Tcyt, abs | 63 (43; 103) | 64 (48; 106) | 74 (36; 100) |
| CM Tcyt, abs | 23 (16; 51) | 28 (21; 39)* | 17 (12; 29) |
| EM Tcyt, abs | 112 (67; 215) | 104 (61; 166) | 97 (28; 146) |
| TEMRA Tcyt, abs | 242 (173; 389)*, + | 169 (95; 312)+ | 191 (87; 224) |
| Th, abs | 822 (654;1145) | 1020 (807;1173) | 844 (590; 986) |
| Naïve Th, abs | 245 (146; 332)+ | 390 (250; 574)+ | 328 (85;475) |
| CM Th, abs | 363 (239;420) | 320 (251; 442) | 271 (203; 336) |
| EM Th, abs | 176 (119; 269) | 196 (151; 256) | 208 (92; 232) |
| TEMRA Th, abs | 21 (10; 48) | 18 (15; 33) | 31 (16; 44) |
| Tregs, abs | 49 (37; 67)+ | 61 (50; 76)+ | 57 (36; 84) |
| Naïve Tregs, abs | 11 (7; 15)+ | 17 (10; 22)+ | 18 (9; 24) |
| CM Tregs, abs | 31 (27;41) | 34 (26; 41) | 30 (20; 44) |
| EM Tregs, abs | 5 (4; 8) | 7 (4; 11) | 8 (4; 10) |
| TEMRA Tregs, abs | < 1 | < 1 | < 1 |
| Double negative (DN) T cells, % | 2.14 (1.41; 4.47) | 2.61 (1.56; 4.23) | 2.84 (1.68; 4.03) |
| DN T cells, abs | 41 (24; 97) | 56 (36; 82) | 47 (26; 70) |
| CD4+CD8dim, % | 0.69 (0.29; 1.24) | 0.47 (0.29; 0.68) | 0.57 (0.23; 1.49) |
| CD4+CD8dim, abs | 11 (4; 25) | 10 (6; 16) | 7 (4; 20) |
| CD8+CD4dim, % | 0.35 (0.22; 0.82) | 0.45 (0.27; 0.81) | 0.40 (0.24; 0.58) |
| CD8+CD4dim, abs | 7 (4; 15) | 9 (6; 16) | 6 (4; 14) |

Comments:

* p<0.01, patient group vs. HV comparison

+ p<0.05, SAS vs. MAS patient comparison
