# Supplementary material for: CD73 Rather Than CD39 Is Mainly Involved in Controlling Purinergic Signaling in Calcified Aortic Valve Disease
Source: Front Genet. 2019 Jul 25;10:604. doi: 10.3389/fgene.2019.00604 (PMC6669234; doi:10.3389/fgene.2019.00604)
Supplement: Supplementary file 2 [file Presentation_1.pdf]

Supplementary Figure 1. Flow cytometry gating strategy applied to identify peripheral blood T cell subsets as well as assess major differentiation stages and subsequent expression of ectonucleotidases CD39 and CD73.

(A) Lymphocytes were selected based on the side scatter (SSC) versus CD45 expression plot by gating on “CD45++”. (B) Discrimination of lymphocyte doublets, single T cell gating based on FSC H versus FSC A (the region “sgl LY” is set on single cells); (C) All lymphocytes were gated on the side scatter/forward scatter plot with a gate “LY”; (D) T cells were identified within total lymphocyte population as “CD3+”; (E) Th (CD3+CD4+) and cytotoxic T cells (CD3+CD8+) were identified within CD3-positive cell population based on CD4 and CD8 expression, respectively; (F) Regulatory T cells (Tregs) were identified as CD4+CD25hi subset within total LY population; (G) Dot plot depicting expression of CD45R0 and CD62L markers on Th cells: naïve Th cells – CD45R0–CD62L+, central memory cells – CD45R0+CD62L+, effector memory cells –CD45R0+CD62L–,effector or “terminally-differentiated” Th cells – CD45R0–CD62L– (also used to identify the same differentiation stages for cytotoxic T cells); (H) Dot plot depicting expression of CD45R0 and CD62L markers on Tcyt cells; (I) Dot plot depicting expression of CD45R0 and CD62L markers on Treg cells; (J), (K) and (L) expression of CD39 and CD73, respectively, by total CM Th, Tcyt, Treg cells (also used with the same position of quadrant gates to assess expression of CD39 and CD73 on naïve, CM, EM and TEMRA Th, Tcyt and Treg subsets).

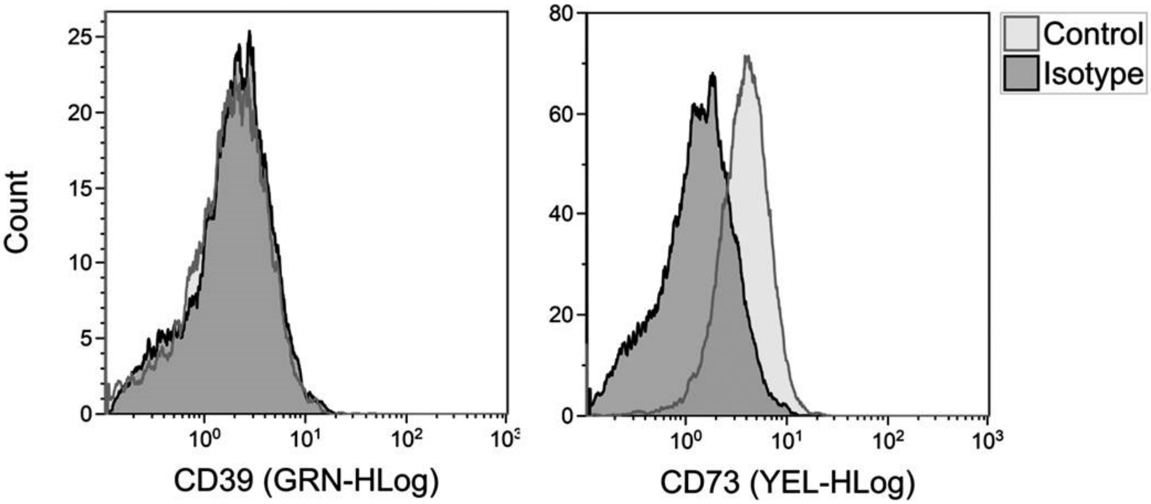

Supplementary Figure 2.

2A. Histogram overlaying staining with isotype-match control and unstimulated control cells stained with anti-CD39 (a) and anti-CD73 (b) antibodies.

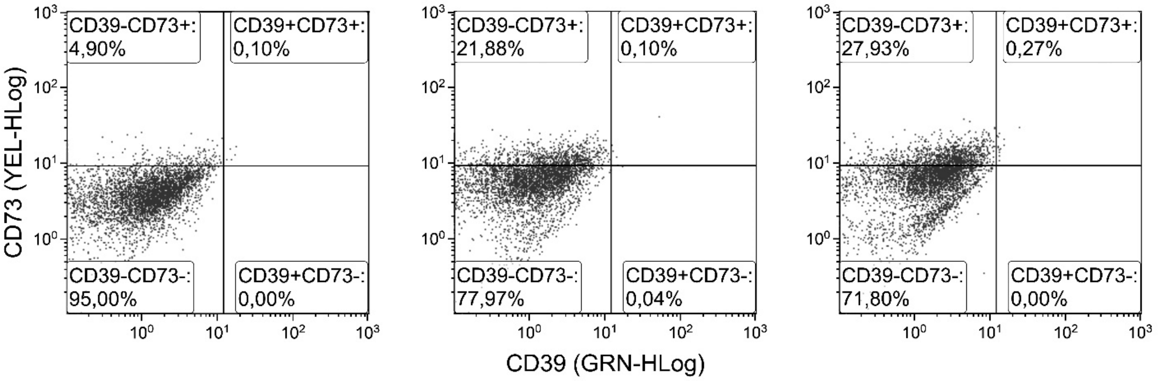

2B. Two-parameter histograms depicting CD39 and CD73 staining of unstimulated (a), adenosine-stimulated (b), and ATP-stimulated (c) cells (7 days after the onset).
